# Liver fat fraction negatively correlates with lumbar BMD in normal body mass index postmenopausal women: mediated by bone marrow fat

**DOI:** 10.3389/fendo.2026.1900687

**Published:** 2026-09-09

**Authors:** Lei Gao, Xiaona Zhu, Ying Liu, Miao Miao, Yongli Zheng, Yan Wang, Ning Zheng, Wei Zhang, Ping Zhang, Jian Zhao

**Affiliations:** 1 Department of Medical Imaging, Hebei Medical University Third Hospital, Shijiazhuang, China; 2 Endocrinology Department, Hebei Medical University Third Hospital, Shijiazhuang, China; 3 Clinical & Technical Support, Philips Healthcare, Wuhan, China; 4 Department of Radiology, Beijing Geriatric Hospital, Beijing, China

**Keywords:** bone mineral density, fat fraction, liver, lumbar, postmenopausal women, proton density fat fraction

## Abstract

**Background:**

The liver-bone axis plays a key role in lipid metabolism and endocrine regulation. However, the relationship between fatty liver disease and both the level and longitudinal changes of bone mineral density (BMD) remains inconclusive. This study aimed to explore the correlation of liver fat with lumbar BMD and bone marrow fat in postmenopausal women.

**Methods:**

In this cross-sectional study, conducted between May 2023 and December 2025, 118 postmenopausal women were included and stratified into 2 groups based on their BMI. All participants underwent quantitative computed tomography (QCT) to assess lumbar BMD and liver fat fraction, and underwent MRI using the mDixon-Quant sequence to assess lumbar proton density fat fraction (PDFF). Correlation analysis, linear regression, and mediation analysis were performed to evaluate the relationships among liver fat, lumbar PDFF, and BMD. Logistic regression analysis was used to assess the impact of liver fat on osteoporosis.

**Results:**

In the normal-BMI group, the liver fat fraction was correlated negatively with BMD [β = -0.368, 95% confidence interval (CI): –0.605 to –0.131, P = 0.003] and positively with lumbar PDFF (β = 0.335, 95% CI: 0.064 to 0.607, P = 0.016) after adjusting for age BMI, and visceral fat area. The liver fat fraction was also identified as an associated factor for osteoporosis [odds ratio (OR) = 1.345, 95% CI: 1.065 to 1.699, P = 0.013] in the normal-BMI group. Mediation analysis demonstrated that lumbar PDFF played a crucial intermediary role (38.6%) in the relationship between liver fat fraction and BMD. However, in the higher BMI group, no correlation was found between liver fat and BMD or lumbar PDFF.

**Conclusion:**

Liver fat fraction is independently associated with lower lumbar BMD in normal-BMI postmenopausal women, partially mediated by bone marrow fat, suggesting it may serve as a potential extra-osseous imaging biomarker for osteoporosis risk.

## 1 Introduction

With the global aging population, an increasing number of women are entering the postmenopausal phase, and the health challenges they face are becoming more pronounced (1). Postmenopausal women are particularly susceptible to osteoporosis and metabolic dysfunction-associated fatty liver disease (MAFLD)–two highly prevalent conditions that may share common pathogenic mechanisms (2–6). In addition, the liver–bone axis plays a key role in lipid metabolism and endocrine regulation (7–9). However, the relationship between fatty liver disease and both the level and longitudinal changes of bone mineral density (BMD) in patients with MAFLD remains inconclusive (10). A cross-sectional study of lumbar bone samples from postmenopausal women with MAFLD reported impairments in trabecular and cortical microstructure of lumbar vertebrae, which may be associated with alterations in bone marrow fat (11). In contrast, another study reported that remarkable associations between NAFLD and both BMD and compressive strength disappeared after adjustment for body mass index (BMI) and visceral fatty tissue (VAT) (12).

Quantitative computed tomography (QCT) can accurately assess lumbar BMD independent of confounding factors such as scoliosis, abdominal aortic sclerosis, vertebral hyperplasia, and other influencing factors. It can simultaneously assess liver fat and VAT without additional radiation exposure (13, 14). The liver fat fraction measured by QCT has been confirmed to correlate well with biochemical lipid extraction (15). Additionally, the proton density fat fraction (PDFF) measured using the mDixon-Quant sequence provides a reliable assessment of lumbar vertebral bone marrow fat, with high accuracy and reproducibility across various observers and MRI platforms (16).

In this study, QCT and MRI mDixon-Quant sequence were combined to establish a noninvasive, multimodal imaging approach to evaluate the liver–bone axis. The study aimed to: (1) explore the association between liver fat and lumbar BMD, and the risk of osteoporosis among the postmenopausal women with varied BMI levels; and (2) assess the mediating role of lumbar PDFF in the relationship between liver fat and lumbar BMD.

## 2 Materials and methods

### 2.1 Study population

**Figure 1:**
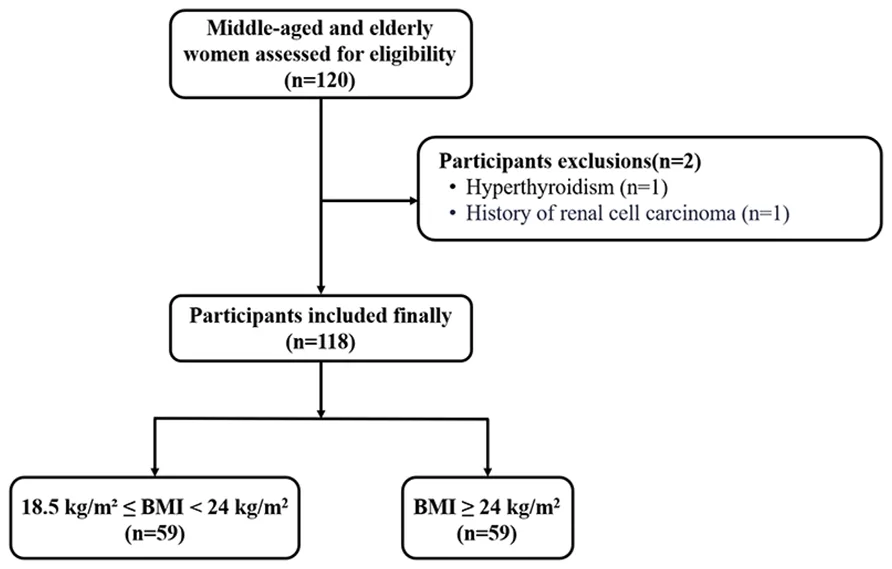
Flowchart of participant selection. BMI, Body mass index.

This study was approved by the Ethics Committee of Hebei Medical University Third Hospital (approval number: W2023-038-1). This study was conducted in accordance with the Declaration of Helsinki and its subsequent amendments. The inclusion criteria for patient selection were as follows: (1) postmenopausal women aged 50 years or older, menopause is deemed to have occurred after 12 consecutive months of amenorrhea (17); and (2) participants underwent both abdominal QCT and lumbar MRI examination on the same day. The exclusion criteria were as follows: (1) history of lumbar fracture, paralysis, or being bedridden; (2) history of hormone replacement therapy or taking medications that affect bone metabolism, such as thiazolidinediones or glucocorticoids; (3) history of diseases influencing bone metabolism, such as hyperthyroidism, liver failure, kidney failure, or tumors; and (4) history of smoking or alcohol abuse. Data on participants’ height, weight, years since menopause, and clinical biochemical laboratory data parameters were collected. Between May 2023 and December 2025, 120 participants who attended the outpatient department of our hospital were initially enrolled. After excluding 2 participants who met the exclusion criteria, 118 middle-aged and elderly women were ultimately included in the study (**Figure 1**).

BMI may affect liver fat fraction, so according to the criteria of the Cooperative Meta-Analysis Group of the Working Group on Obesity in China (18), participants were stratified into 2 subgroups based on BMI: a normal-BMI group (18.5 kg/m^2^ ≤ BMI < 24 kg/m^2^) and a higher-BMI group (BMI ≥ 24 kg/m^2^).

### 2.2 QCT image acquisition and analysis

All participants underwent an abdominal CT scan with QCT in-scan calibration using a 128-slice multidetector CT (Somatom Sensation; Siemens, Erlangen, Germany) with the following parameters: tube voltage = 120 kV; tube current = 125 mAs; pixel size = 0.78 mm^2^; pitch = 0.8 mm; matrix = 512 × 512; scanning field of view (FOV) = 500 mm; and thickness = 1 mm. Scanning was performed in the supine position, from the top of the diaphragm to the lower edge of the third lumbar vertebra. Images were transferred to the QCT workstation (Mindways Software Inc., TX, USA) for measurement of liver fat fraction, visceral fat area (VFA) and lumbar BMD.

**Figure 2:**
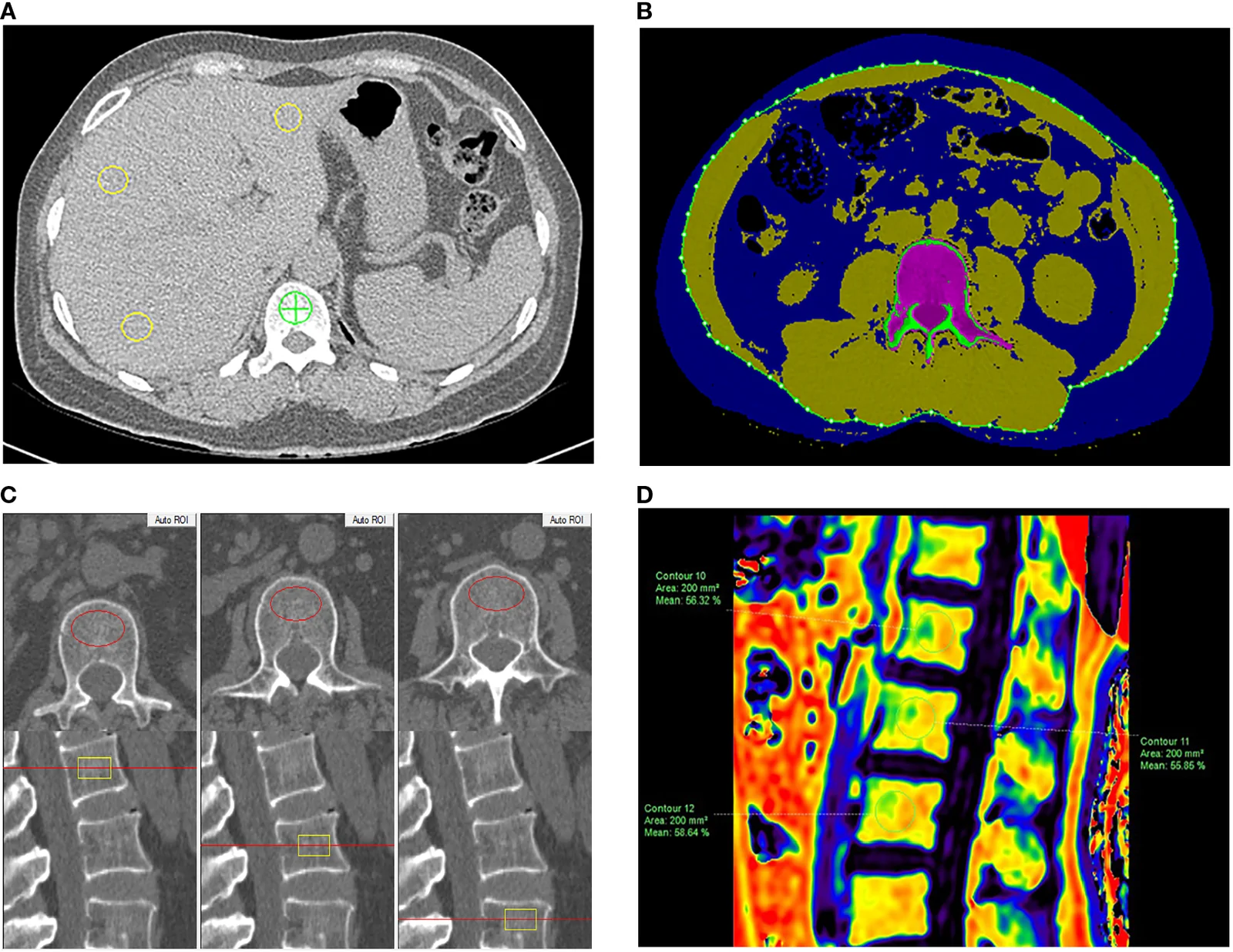
Delineation of the ROIs for liver fat fraction **(A)**, VFA **(B)**, lumbar BMD **(C)**, and lumbar PDFF **(D)**. ROI, regions of interest; VFA, visceral fat area; BMD, Bone mineral density; PDFF, proton density fat fraction.

A previous study has demonstrated that liver fat measured by QCT exhibits good correlation and accuracy with PDFF measured by chemical shift–encoded MRI (19). Liver fat fraction was measured by manually placing three 300 mm² ROIs in the left, right anterior, and right posterior hepatic lobes at the main portal vein bifurcation, avoiding vessels and bile ducts (**Figure 2A**). VFA was measured using semi-automated delineation at the mid-L3 level (**Figure 2B**). The mean value of these measurements was calculated to represent the liver fat fraction. Lumbar BMD was measured by delineating ROIs at the middle level of the L1-L3 vertebrae, avoiding bone cortex and hyperplasia, and the average BMD of the three vertebrae was calculated (**Figure 2C**).

### 2.3 MRI acquisition and analysis

Lumbar spine were examined using the mDixon-Quant sequence on a 3.0-Tesla MRI Scanner (Ingenia CX; Philips). Parameters was: repetition time (TR) = 8 milliseconds; echo time (TE) = shortest; flip angle = 3°; FOV = 160 × 298 × 160 mm; voxel = 2.5 × 1.5 × 5.0 mm^3^; slice thickness = 5 mm; number of signal averaged = 2; and acquisition time = 54 seconds. PDFF maps (water, fat, T2^*^, R2^*^, PDFF) were reconstructed. ROIs were manually sketched on the PDFF images using Intellispace Portal software (ISP, version 10.1; Philips, The Netherlands). The PDFF values of the L1-L3 vertebrae were obtained by outlining ROIs at the middle level of lumbar 1–3 vertebrae, avoiding osteophytes, cortical bone, and basal vertebral veins. Lumbar PDFF was defined as the average PDFF of L1–L3 (**Figure 2D**).

### 2.4 Statistical analysis

The Kolmogorov–Smirnov test was used to assess the normality of continuous variables. The normally distributed data were presented as mean ± standard deviation, whereas non-normally distributed data were presented as medians with interquartile ranges. The comparisons between 2 groups were performed using independent-sample t tests or the Mann–Whitney U test. Simple correlation analysis and linear regression analyses were used to evaluate the associations between lumbar and hepatic imaging parameters. In the linear regression analyses, BMD and lumbar PDFF were treated as dependent variables in separate models, with liver fat fraction as the independent variable. Due to collinearity between the years since menopause and age, only age, BMI and VFA were included as covariates. Osteoporosis was defined as the average BMD < 80 mg/cm^3^ measured by QCT (20). Logistic regression analysis was conducted to assess the effect of liver fat fraction on osteoporosis risk, adjusting for age, BMI and VFA. The potential mediating role of lumbar PDFF on the relationship between liver fat fraction and BMD was analyzed using the PROCESS macro (Model 4) of SPSS, with age, BMI and VFA adjusted. To determine effect sizes and obtain 95% confidence intervals, we assessed direct, indirect, and total effects using the bootstrap method with 5000 iterations. After excluding obese individuals from the higher BMI group, a sensitivity analysis was conducted. A significant effect was shown when the 95% confidence interval did not include zero. Statistical analyses were conducted using SPSS (version 27.0; SPSS Inc., IL, USA) and R (version 4.4.2; R Foundation for Statistical Computing, Vienna, Austria). A P value <0.05 was considered statistically significant.

## 3 Results

### 3.1 Baseline characteristics of participants

A total of 118 participants with a median age of 62 years (range, 51–81 years) were included in this study and stratified into 2 groups based on BMI: a normal-BMI group (18.5 kg/m² ≤ BMI < 24 kg/m², n = 59) and a higher-BMI group (BMI ≥ 24 kg/m², n = 59). The demographic characteristics, laboratory data, and radiologic imaging parameters of the liver and lumbar are summarized in **Table 1**. No significant differences were observed between the 2 groups in terms of age, alanine aminotransferase (ALT), aspartate aminotransferase (AST), total cholesterol (TC), low-density lipoprotein cholesterol (LDL-C), BMD, and lumbar PDFF (all P > 0.05) (**Table 1**).

**Table 1:** Demographic characteristics, biochemical and radiological imaging parameters of the participants.

Variables	Total (n=118)	Normal BMI (n=59)	Higher BMI (n=59)	T/Z	P
Age (years)	62.71 ± 7.06	62.56 ± 7.33	62.86 ± 6.84	-0.23^†^	0.816
Years at menopause (years)	49.56 ± 3.50	49.35 ± 3.56	49.76 ± 3.46	-0.64^†^	0.522
Years since menopause (years)	11.00 (6.38, 19.00)	10.00 (7.00,18.00)	12.00 (8.00, 18.50)	0.28^‡^	0.784
BMI (kg/m^2^)	24.14 ± 2.78	22.00 ± 1.44	26.29 ± 2.04	-13.22^†^	<0.001^***^
ALT(U/L)	20.98 ± 9.38	20.04 ± 9.73	21.92 ± 9.00	-1.09^†^	0.278
AST(U/L)	20.99 ± 7.04	20.43 ± 7.87	21.56 ± 6.12	-0.87^†^	0.387
TC (mmol/L)	5.10 ± 0.96	5.24 ± 0.86	4.96 ± 1.05	1.60^†^	0.112
TG (mmol/L)	1.30 ± 0.66	1.16 ± 0.59	1.44 ± 0.70	-2.38^†^	0.019^*^
HDL-C (mmol/L)	1.51 ± 0.28	1.59 ± 0.28	1.42 ± 0.25	3.45^†^	<0.001^***^
LDL-C (mmol/L)	2.88 ± 0.67	2.95 ± 0.58	2.81 ± 0.75	1.14^†^	0.257
VLDL-C (mmol/L)	0.59 ± 0.30	0.53 ± 0.27	0.65 ± 0.32	-2.22^†^	0.028^*^
VFA (cm^2^)	140.63 ± 50.91	117.60 ± 43.19	163.65 ± 47.79	-5.49^†^	<0.001^***^
BMD (mg/cm^3^)	98.71 ± 29.04	102.01 ± 30.39	95.41 ± 27.49	1.24^†^	0.219
Lumbar PDFF(%)	55.67 ± 6.74	55.50 ± 5.79	55.85 ± 7.62	-0.28^†^	0.778
Liver fat fraction (%)	6.50 (4.91, 9.88)	5.63 (4.47, 7.82)	8.07 (5.47, 11.08)	3.13^‡^	0.002^**^

^†^ The independent sample t-test was used. ^‡^The Mann-Whitney U test was used. ^*^P<0.05, ^**^P<0.01, ^***^P<0.001.

BMI, body mass index; ALT, alanine aminotransferase; AST, aspartate aminotransferase; TC, total cholesterol; TG, triglyceride; HDL-C, high density lipoprotein cholesterol; LDL-C, low density lipoprotein cholesterol; VLDL-C, very low-density lipoprotein cholesterol; VFA, visceral fat area; BMD, bone mineral density; PDFF, proton density fat fraction.

Participants in the higher-BMI group had significantly higher VFA (163.65 ± 47.79 vs 117.60 ± 43.19, P <0.001), triglyceride (TG) levels (1.44 ± 0.70 vs 1.16 ± 0.59 mmol/L, P = 0.019) and very low–density lipoprotein cholesterol (VLDL-C) levels (0.65 ± 0.32 vs 0.53 ± 0.27 mmol/L, P = 0.028) but lower high-density lipoprotein cholesterol (HDL-C) levels (1.42 ± 0.25 vs 1.59 ± 0.28 mmol/L, P <0.001) compared with the normal-BMI group. The higher-BMI group demonstrated significantly higher liver fat content (8.07 [5.47, 11.08]% vs 5.63 [4.47, 7.82] %, P = 0.002) than the normal-BMI group (**Table 1**).

### 3.2 Association between lumbar vertebrae and liver imaging parameters

**Figure 3:**
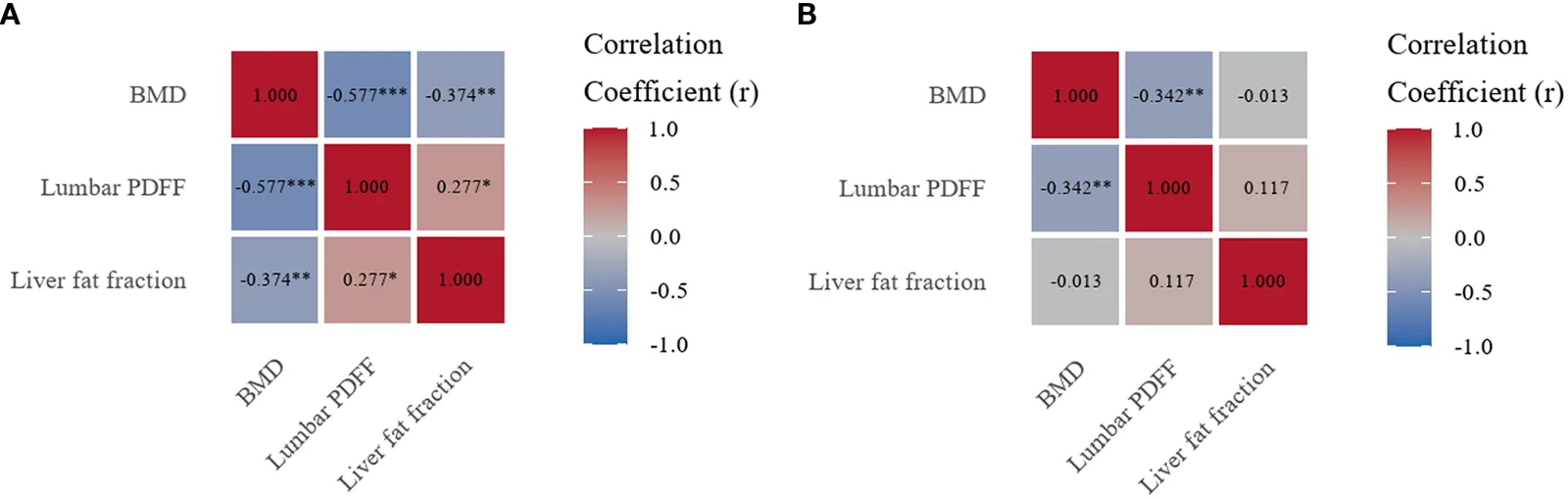
Correlation of heat map among liver fat fraction, lumbar BMD, and lumbar PDFF in the normal-BMI group **(A)** and higher-BMI group **(B)**. BMD, Bone mineral density; PDFF, proton density fat fraction. ^*^P < 0.05, ^**^P < 0.01, ^***^P < 0.001.

Lumbar PDFF demonstrated a significant negative correlation with BMD (normal-BMI group: r = −0.577, P <0.001; higher-BMI group: r = −0.342, P = 0.008). In the normal-BMI group, the liver fat fraction was correlated negatively with BMD (r = −0.374, P = 0.003) and positively with lumbar PDFF (r = 0.277, P = 0.034) (**Table 2**, **Figure 3A**). However, in the higher-BMI group, no significant correlation was observed between liver fat fraction and either BMD or lumbar PDFF (P >0.05) (**Table 2**, **Figure 3B**).

**Table 2:** Correlation analysis between lumbar BMD, PDFF and liver fat fraction.

Group		BMD	Lumbar PDFF	Liver fat fraction
		r/P	r/P	r/P
Normal BMI	BMD	1		
	Lumbar PDFF	-0.577/<0.001^†***^	1	
	Liver fat fraction	-0.374/0.003^‡**^	0.277/0.034^‡*^	1
Higher BMI	BMD	1		
	Lumbar PDFF	-0.342/0.008^†**^	1	
	Liver fat fraction	-0.013/0.922^‡^	0.117/0.377^‡^	1

^†^ Pearson correlation analysis was used. ^‡^Spearman correlation analysis was used. ^*^P<0.05, ^**^P<0.01, ^***^P<0.001.

BMI, body mass index; BMD, bone mineral density; PDFF, proton density fat fraction.

**Figure 4:**
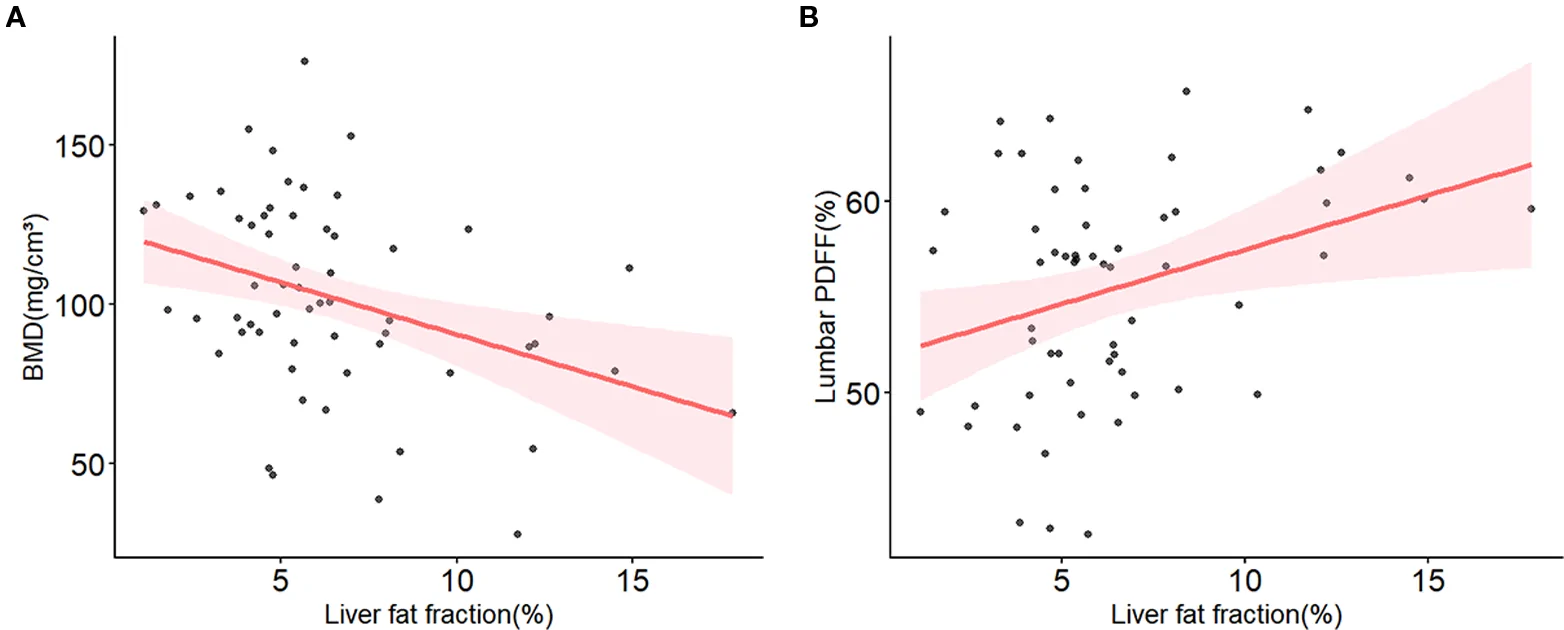
Linear regression analysis graphs of the relationships of liver fat fraction with lumbar BMD **(A)** and lumbar PDFF **(B)**, adjusted for age, BMI and VFA in the normal-BMI group. Light pink areas represent 95% confidence intervals. BMD, Bone mineral density; BMI, body mass index; VFA, visceral fat area; PDFF, proton density fat fraction.

In the normal-BMI group, the liver fat fraction was correlated negatively with BMD (β = −0.368, 95% CI: –0.605 to –0.131, P = 0.003) and positively with lumbar PDFF (β = 0.335, 95% CI: 0.064 to 0.607, P = 0.016), after adjusting for age, BMI and VFA (**Figures 4A, B**). No significant correlations were observed between liver fat fraction and lumbar BMD or PDFF in the BMI ≥24 kg/m^2^ group (all P >0.05) (**Tables 3**, **4**).

**Table 3:** The association between lumbar BMD and liver fat fraction.

Group	Model 1			Model 2		
	Std β 95%CI	T	P	Std β 95%CI	T	P
Normal BMI	-0.378 (-0.623 to -0.132)	-3.080	0.003^**^	-0.368 (–0.605 to –0.131)	-3.112	0.003^**^
Higher BMI	-0.019 (-0.284 to 0.247)	-0.140	0.889	0.190 (-0.087 to 0.467)	1.374	0.175

Model 1: no covariates were adjusted. Model 2: Age, BMI and visceral fat area at the L3 level were adjusted. The dependent variable was bone mineral density, and the independent variable is the liver fat fraction.

BMD, bone mineral density; BMI, body mass index. ^**^P<0.01.

**Table 4:** The association between lumbar PDFF and liver fat fraction.

Group	Model 1			Model 2		
	Std β 95%CI	T	P	Std β 95%CI	T	P
Normal BMI	0.333 (0.083, 0.583)	2.669	0.010^*^	0.335 (0.064, 0.607)	2.476	0.016^*^
Higher BMI	0.095 (-0.169 to 0.359)	0.719	0.475	-0.128 (-0.411 to 0.155)	-0.907	0.368

Model 1: no covariates were adjusted. Model 2: Age, BMI and visceral fat area at the L3 level were adjusted. The dependent variable was lumbar PDFF, and the independent variable is the liver fat fraction.

PDFF, proton density fat fraction; BMI, body mass index. ^*^P<0.05.

**Figure 5:**
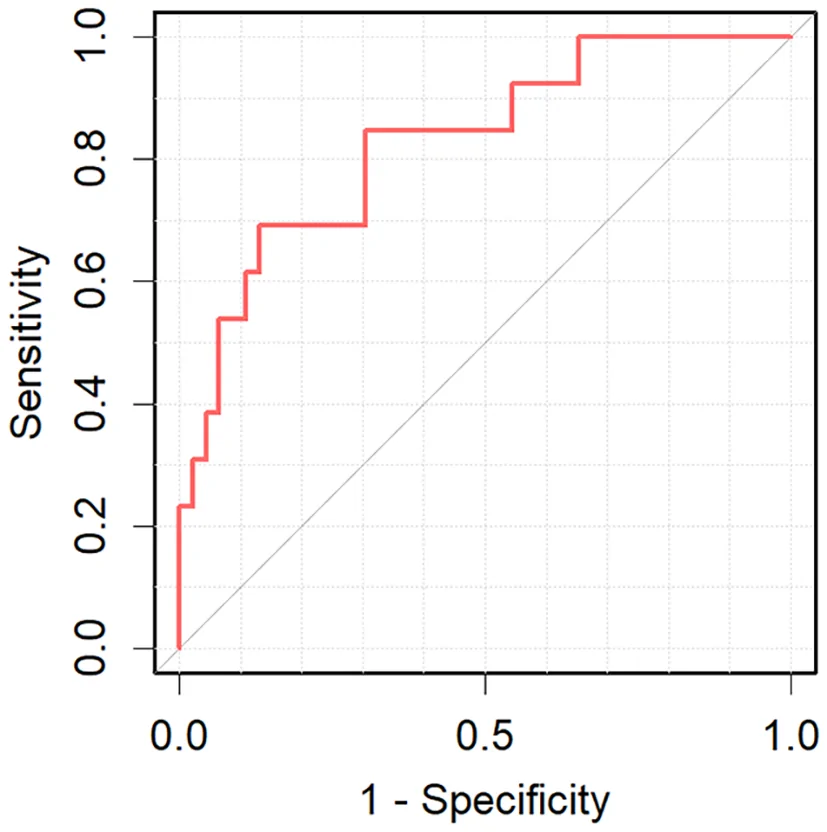
ROC curve of liver fat fraction for predicting osteoporosis in the normal BMI group. The predicted probability was derived from a logistic regression model adjusting for age, BMI, and VFA. The AUC was 0.828 (95% CI: 0.698–0.958). ROC: receiver operating characteristic; BMI, body mass index; VFA, visceral fat area.

In the normal-BMI group, the liver fat fraction was identified as an independent associated factor for osteoporosis [odds ratio (OR) =1.345, 95% CI: 1.065 to 1.699, P = 0.013]. After adjustment for age, BMI, and VFA, the area under the ROC curve (AUC) of liver fat fraction for predicting osteoporosis was 0.828 (95% CI: 0.698–0.958). The optimal cutoff value of the predicted probability derived from the multivariable logistic regression model after adjustment for age, BMI, and VFA was 0.293, with a sensitivity of 69.2% and a specificity of 87.0% (**Figure 5**). However, in the higher-BMI group, the liver fat fraction was not significantly associated with osteoporosis. The logistic analysis demonstrated no statistically significant association between the liver fat fraction and risk of osteoporosis in the higher-BMI group (OR = 1.050, 95% CI: 0.913 to 1.206, P = 0.494).

**Figure 6:**
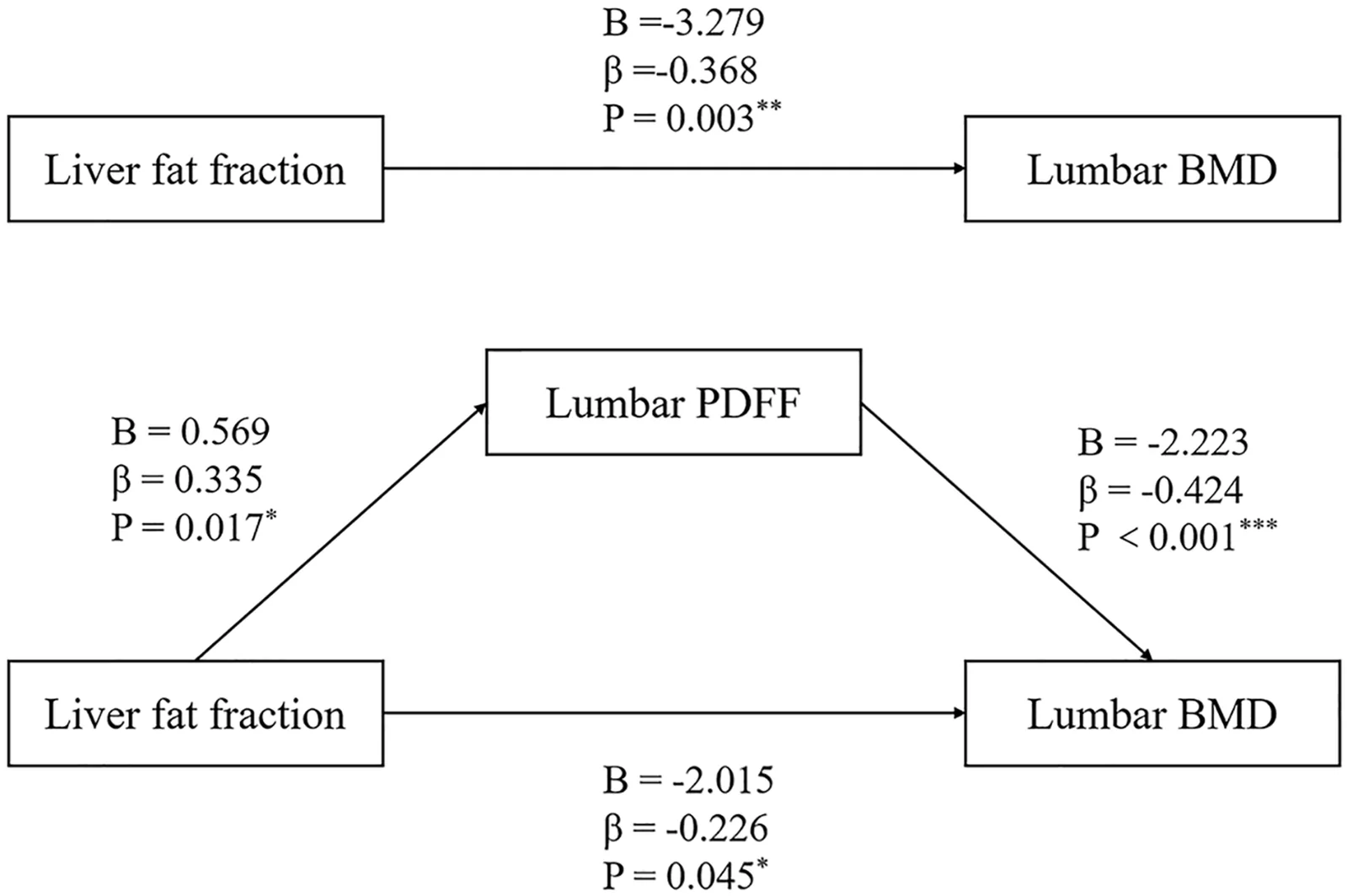
Mediation analysis displaying the contribution of lumbar PDFF to the relationship between the liver fat fraction and lumbar BMD, adjusted for age, BMI, and VFA. BMD, Bone mineral density; BMI, body mass index; PDFF, proton density fat fraction; VFA, visceral fat area.

The mediation analysis indicated that lumbar PDFF accounted for 38.6% (95% CI: 0.089 to 0.891) of the association between liver fat fraction and lumbar BMD (indirect effect: −1.264, 95% CI: −2.536 to −0.305; total effect: −3.279, 95% CI: −5.391 to −1.167) in the normal-BMI group, after adjusting for age, BMI and VFA(**Figure 6**). However, in the higher-BMI group, the mediating effect of lumbar PDFF on the relationship between liver fat fraction and lumbar BMD was not statistically significant (indirect effect: −0.168; 95% CI: −0.703 to 0.396). The total effect of liver fat fraction on lumbar BMD was also non-significant (B = -0.390; 95% CI: -2.032 to 1.253), as was the direct effect after accounting for lumbar PDFF (B = -0.222; 95% CI: -1.858 to 1.414). After excluding individuals with BMI ≥ 28 kg/m² from the higher BMI group, the mediation effect of lumbar PDFF on the association between liver fat and BMD remained non-significant (indirect effect: −0.148; 95% CI: −0.808 to 0.590).

## 4 Discussion

The main findings of this study were as follows: in postmenopausal women with normal BMI, the liver fat fraction was correlated negatively with lumbar BMD and positively with lumbar PDFF. The liver fat fraction was also identified as a risk factor for osteoporosis in this group. The lumbar PDFF significantly mediated the negative association between liver fat and BMD, accounting for 38.6% of the total effect. In contrast, in postmenopausal women with higher BMI (≥24 kg/m²), no significant association was observed between liver fat fraction and lumbar BMD or PDFF.

In the normal-BMI group of this study, the observed positive correlation between liver fat and lumbar PDFF, along with the negative correlation between liver fat and BMD, suggests that hepatic lipid metabolism may influence bone homeostasis through the “liver–bone axis.” Although the liver and the bone are physically distant, the liver can secrete hepatokines, such as bone morphogenetic protein 9, fibroblast growth factor 21, and insulin-like growth factor 1, which play key roles in regulating bone metabolism (7). Conversely, osteokines such as osteocalcin, sclerostin, and osteopontin also regulate liver metabolism through the endocrine system (7). Furthermore, our results demonstrated that liver fat was a risk factor for osteoporosis. After adjustment for age, BMI and VFA, each one-unit increase in the liver fat fraction corresponded to approximately 34.5% higher odds of osteoporosis. An increase in the liver fat content may promote liver inflammation and fibrosis, disrupting metabolic homeostasis and increasing the risk of osteoporosis (21, 22). Consequently, postmenopausal women with normal BMI who have fatty liver disease may be considered at an elevated risk for osteoporosis.

Consistent with previous studies (10, 23), our study indicated that in postmenopausal women with a BMI ≥24 kg/m², no significant associations were observed between liver fat and both lumbar BMD and PDFF. We speculated that metabolic disorders in higher BMI (≥ 24 kg/m²) individuals may mask the direct effects of liver fat on bones. Additionally, the increased mechanical load on the bones in these individuals can stimulate osteoblast activity, and hyperestrogenemia resulting from elevated aromatase activity in abundant adipose tissue may further contribute to higher BMD (24, 25). A Mendelian randomization study revealed that higher BMI causally increases BMD, with this effect consistently observed in both East Asian and European populations (26). We speculated that in individuals with higher BMI, the skeletal protective effect of elevated BMI may obscure or counteract any potential negative impact of liver fat on BMD. Furthermore, in people with higher BMI, multiple metabolic disorders (such as insulin resistance, systemic chronic inflammation, and dyslipidemia) often coexist (27). In this context, increased liver fat represents only one link of a complex metabolic disorder network. Bone health may have already been damaged by other stronger factors, such as insulin resistance, rendering the influence of liver fat statistically insignificant.

Another finding of this study is that lumbar bone marrow fat mediates the “liver–bone axis.” Mediation analysis indicated that in normal postmenopausal women, 38.6% of the negative impact of liver fat on BMD was mediated by increasing the lumbar bone marrow fat. This finding provides an imaging-based perspective for understanding the interplay between the liver and bone. Previous studies have confirmed a clear negative correlation between bone marrow fat fraction and BMD (28, 29). Bone marrow fat content is inversely related to BMD, reflecting the displacement of lost bone tissue by adipose deposits (30). Liver steatosis has been associated with an increase in bone marrow adipose tissue, which, in turn, correlates with a decrease in BMD (31). Considering that elevated bone marrow fat is linked to osteoporosis, this study suggests that the liver may influence BMD through metabolic pathways independent of obesity, thereby contributing to increased osteoporosis risk in normal BMI postmenopausal women.

Previous studies have reported inconsistent findings regarding the relationship between liver fat and BMD. A study involving 4264 Koreans demonstrated that a high fatty liver index correlated with BMD in men but not in women (32). In contrast, a meta-analysis reported that NAFLD was associated with reduced BMD and an increased risk of osteoporosis or osteoporotic fractures (33). Additionally, Akın Abbasoğlu et al. demonstrated a significant correlation between the liver fat fraction and lumbar bone marrow in women (34). We propose that differences in BMI distribution across study populations could be a key source of heterogeneity.

This study had several limitations. First, as this was a cross-sectional study, the causal relationship between liver fat and BMD could not be established. Second, the study population included only postmenopausal women, which might limit the generalizability of the conclusions. Third, the absence of bone turnover markers (such as C-terminal telopeptide of type I collagen and Procollagen type I N-terminal propeptide) and hormone assays (e.g., FSH, estradiol) limited the depth of exploration of the molecular mechanisms of liver–bone interaction and precluded further refinement of menopausal status classification. Fourth, the relatively small sample size of this study may have affected the statistical power to some extent. Future studies should incorporate molecular and biologic indicators, along with a prospective design with a larger sample size, to elucidate the mechanism underlying liver–bone interaction.

## 5 Conclusions

This study demonstrated differences in the liver–bone associations across BMI stratifications using multimodal imaging techniques. The findings indicate that in postmenopausal women with normal BMI, the liver fat fraction is a risk factor for reduced BMD and osteoporosis, with bone marrow adipose tissue playing a significant mediating role in this relationship. These results highlight the potential clinical value of liver fat quantification by QCT as an opportunistic imaging biomarker for osteoporosis risk stratification in postmenopausal women with normal BMI. As liver fat fraction can be derived from abdominal CT data, this approach may facilitate opportunistic identification of individuals at higher osteoporosis risk without additional imaging, thus enabling early intervention.

## Data Availability

The raw data supporting the conclusions of this article will be made available by the authors, without undue reservation.
